# Quality of a fished resource: Assessing spatial and temporal dynamics

**DOI:** 10.1371/journal.pone.0196864

**Published:** 2018-06-06

**Authors:** Sarah J. Teck, Julio Lorda, Nick T. Shears, Tal Ben-Horin, Rebecca E. Toseland, Sarah T. Rathbone, Dave Rudie, Steven D. Gaines

**Affiliations:** 1 Department of Ecology, Evolution, and Marine Biology, University of California, Santa Barbara, California, United States of America; 2 Tijuana River National Estuarine Research Reserve, Imperial Beach, CA, United States of America; 3 Facultad de Ciencias, Universidad Autónoma de Baja California, Ensenada, Baja California, México; 4 Leigh Marine Laboratory, Institute of Marine Science, University of Auckland, Auckland, New Zealand; 5 College of the Environment and Life Sciences, University of Rhode Island, Kingston, RI, United States of America; 6 Stanford Institute for Economic Policy Research, Stanford University, Stanford, CA, United States of America; 7 Community Seafood, Goleta, CA, United States of America; 8 Bren School of Environmental Science and Management, University of California, Santa Barbara, California, United States of America; 9 Catalina Offshore Products, San Diego, CA, United States of America; California Polytechnic State University, UNITED STATES

## Abstract

Understanding spatio-temporal variability in the demography of harvested species is essential to improve sustainability, especially if there is large geographic variation in demography. Reproductive patterns commonly vary spatially, which is particularly important for management of “roe”-based fisheries, since profits depend on both the number and reproductive condition of individuals. The red sea urchin, *Mesocentrotus franciscanus*, is harvested in California for its roe (gonad), which is sold to domestic and international sushi markets. The primary driver of price within this multi-million-dollar industry is gonad quality. A relatively simple measure of the fraction of the body mass that is gonad, the gonadosomatic index (GSI), provides important insight into the ecological and environmental factors associated with variability in reproductive quality, and hence value within the industry. We identified the seasonality of the reproductive cycle and determined whether it varied within a heavily fished region. We found that fishermen were predictable both temporally and spatially in collecting urchins according to the reproductive dynamics of urchins. We demonstrated the use of red sea urchin GSI as a simple, quantitative tool to predict quality, effort, landings, price, and value of the fishery. We found that current management is not effectively realizing some objectives for the southern California fishery, since the reproductive cycle does not match the cycle in northern California, where these management guidelines were originally shaped. Although regulations may not be meeting initial management goals, the scheme may in fact provide conservation benefits by curtailing effort during part of the high-quality fishing season right before spawning.

## Introduction

Quality plays an important role in the price of all fish products, especially when the product is served raw. High-grade fresh fish can be worth 4 to 20 times the price of lower-grade fish [1,2]. The manner in which a fish is caught, handled, and stored affects quality and thus price [3–5]. In addition, quality is also often related to a species’ reproductive cycle. For example, in several fisheries (sea urchin, scallop, herring, sturgeon, squid, and salmon) quality peaks before the spawning season [3,6–10]. Reproductive condition can vary across seasons, years, and regions due to many environmental and ecological factors, such as resource availability and quality, spawning or nursery habitat availability and quality, temperature, climate, and upwelling regime [3,11–21]. Understanding how reproduction in a marine resource varies can not only inform population models but also can provide insight into the value of a fished product.

When a fishery is roe-based and the product is served raw, such as sea urchins, there can be a high variability in quality due to the reproductive state of the organism and thus a high variability in product price [22,23]. In recent decades, hundreds of millions of pounds of red sea urchins have been hand-collected by commercial fishermen diving in California’s coastal waters (California Department of Fish and Wildlife [CDFW] data www.wildlife.ca.gov/Conservation/Marine/Invertebrates/Sea-Urchin). This multi-million-dollar industry relies on a consistent, fresh product and is marketed as the sushi product *uni* (see S1 Appendix for more details). The principal sea urchin species exploited in California is the red sea urchin, *Mesocentrotus franciscanus* (previously *Strongylocentrotus* A. Agassiz, 1863; see [24]). Once sea urchin divers bring their catch to shore, the gonads are typically processed, packaged into boxes, and shipped overnight to buyers.

Knowledge about spatial and temporal variability in sea urchin reproduction has been used to inform management, resulting in seasonal closures that serve to limit harvest during a particular reproductive season. For instance in Japan, Chile, and Baja California, Mexico the sea urchin fisheries are closed during the spawning seasons [25,26]. Within Japan and Chile these spawning seasons and thus closure periods vary in timing and duration among regions; in Japan there are up to 14 different management zones for two sea urchin species [26]. Rather than setting fishing limits based on spawning period, the seasonal management scheme in California was modeled after the state of Washington’s fishery, where harvest was closed during the season with low gonad quality. The rationale was that it would be economically advantageous to limit effort during the period of lowest gonad yield and depressed value (P. Kalvass, pers. comm.). The California Department of Fish and Wildlife (CDFW) partially based California’s state-wide seasonal regulations on data from a two-year period of red sea urchin processor gonad and price data from northern California (P. Kalvass, pers. comm.). Statewide in California the limited fishing season, originally set in 1990, was during May through September, and the northern California fishery was also closed in July, the month of lowest gonadosomatic index (GSI; i.e., the ratio of wet gonad weight to wet whole weight) [27]. When these regulations went into place initially, members of the sea urchin fishing industry in southern California agreed with this reduction in fishing days in the summer due to a diminished Japanese demand and to prevent oversupply during this favorable-weather season (D. Rudie, pers. obs.). Managers in California advocated a complete summer closure, when gonad quality, size, and thus prices were considered low. However, managers compromised with members of the industry, who wanted to avoid a long industry closure to keep an active market and skilled labor employed in the processing companies, and instead established seasonal fishing-day limitations (P. Kalvass, pers. comm. and D. Rudie, pers. obs.). Currently across the entire state of California the sea urchin fishery is limited to four days per week from June through October and is open the rest of the year (California Code of Regulations § 120.7, Title 14, 2008 Amendment). It is important to note that limiting effort during the period of depressed value may be ineffective, if effort is already low during this season. Effort limitations could be placed during the time of year when effort is likely to be highest, and in California this is the reproductive season of highest gonad yield and prices.

A key question is whether a single seasonal regulation makes sense for a state with such diverse ecological regions, spanning two marine provinces [28]. The majority of the state’s red sea urchin landings originate from the northern Channel Islands area in Southern California, however regulations were based on the reproductive dynamics of sea urchins in Northern California (nearly 800 kilometers away) and may be inappropriate for Southern California. The Port of Fort Bragg in Northern California ranks third for cumulative commercial sea urchin landings since the start of the fishery, accounting for approximately 13% of the state’s landings, however landings originating from the northern Channel Islands region (into the Ports of Santa Barbara and Oxnard, ranking first and second, respectively) account for approximately 47% of the state’s cumulative landings since the start of the fishery (CDFW data). We therefore focused on the fishery within the northern Channel Islands in this study to evaluate seasonal reproductive dynamics and seasonal fishing patterns. Our research investigated three objectives: (1) to evaluate the spatial and seasonal dynamics of the red sea urchin reproductive cycle and its relationship with industry-quality estimates and price; (2) to assess fishing patterns across the regulatory time periods, regions, and seasons; and (3) to quantify how fishermen respond to urchin reproductive dynamics, relating fishing effort, landings, price, and value to spatial and temporal variation in red sea urchin reproductive condition.

In the ecological literature, reproductive condition (i.e., a proxy of potential reproductive output) is often measured as gonadosomatic index (GSI) [29]. This metric is simple and objective; it can be easily measured in a laboratory, boat, or dock. Furthermore, it is quantitative, as opposed to the qualitative processor grading scale that is typically employed by buyers in the industry. Gonadosomatic index is predictable across the various stages in the reproductive cycle [21,29], so it is a simple way to compare demographics among seasons and locations.

Although California sea urchin are fished year round, the price differential paid for sea urchin roe across varying reproductive stages can be substantial [22,27], which creates a strong incentive for selectively harvesting in the best locations and at the best times during the year. Urchins increase in gonad size due to the growth of nutritive phagocytes (NPs) [22,30], for red sea urchins this occurs during the summer as they consume abundant drift kelp. Then these NPs support the growth and development of the germ cells (GCs) just before and during the spawning season [22,30,31]. Typically, urchins spawn just after they reach their peak in gonad size. The reproductive “ripeness,” or fully mature gonads at the end of gametogenesis, occurs during the spawning season when most of the nutritive phagocytes have shrunken [22]. This is the season when sushi quality declines as GSI declines. Spawning generally indicates lower quality to processors, since most consumers do not like the grainy, watery texture of spawning gonads [22,32]. The gonad reaches its maximum size (highest GSI) just before spawning begins and subsequently shrinks as more gametes are released [17,22,33,34]. Once a sea urchin has fully spawned, the gonad is not as sweet and is smaller (with the lowest GSI) but has a firmer texture, which is preferred by the industry due to the more lasting quality of the product, or “shelf life” (D. Rudie, pers. obs.). In this study, we investigate whether GSI can also be an effective indicator of gonad quality, and thus the quality component of price. Although price may reflect the seasonal supply and demand, the grade of a sea urchin has a large influence on its price. When grading the quality of sea urchin for sushi, processors consider size and several qualitative measures, such as taste, shape, color, texture, and firmness (see S1 Appendix for more details on sushi grades; [22]). If GSI is a good proxy for the industry’s quality metric, it can be used as a quantitative measure to predict the potential seasonal value of sea urchins.

## Materials and methods

### Red sea urchin seasonal reproductive cycle

**Fig 1:**
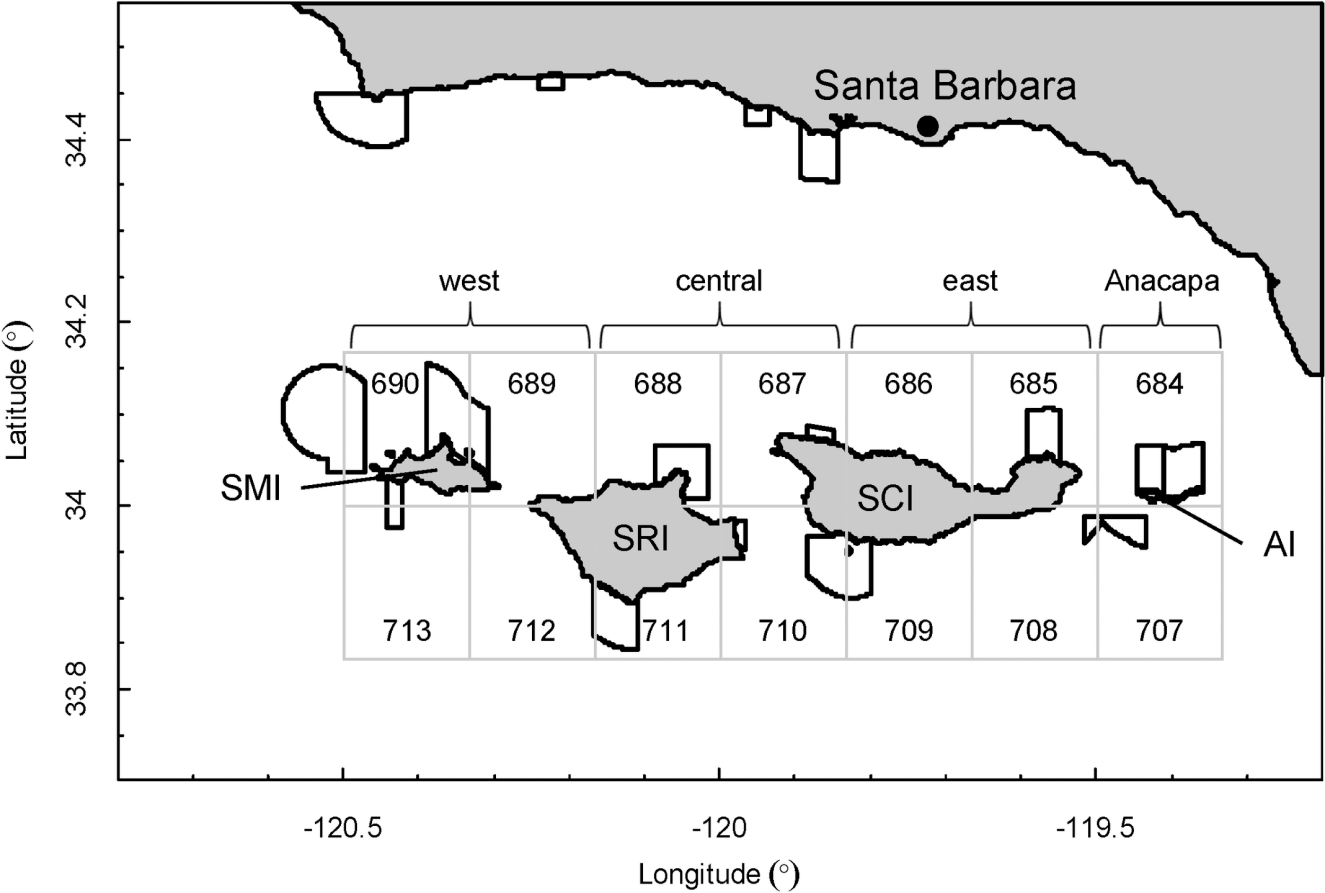
Map of the Northern Channel Islands. Map of the Santa Barbara Channel with California Department of Fish and Wildlife (CDFW) 10 x 10 nautical mile fishing blocks surrounding the northern Channel Islands from west to east: San Miguel Island (SMI), Santa Rosa Island (SRI), Santa Cruz Island (SCI), and Anacapa Island (AI). All subregions include 4 CDFW 10 x 10 nautical mile blocks, except Anacapa only includes 2 blocks: *west* includes all of SMI and the western tip of SRI, *central* includes the majority of SRI and the western tip of SCI, *east* includes the majority of SCI, and *Anacapa* includes the entire island of Anacapa. Marine reserves are outlined in black.

Our first objective focused on the evaluation of the annual reproductive cycle for sea urchins and how it varied across the northern Channel Islands. To examine spatial and seasonal variability in red sea urchin reproductive condition, we sampled catch from commercial fishermen at the port of Santa Barbara (34°24'16.4"N 119°41'32.8"W) approximately once per month from December 2008 to December 2011. We purchased between 10 and 30 haphazardly selected red sea urchins per haphazardly selected boat per sampling date (total *n* = 2759 urchins). All sea urchins were harvested from San Miguel Island (SMI), Santa Rosa Island (SRI), and Santa Cruz Island (SCI) (Fig 1).

Gonadosomatic index (GSI) reflects the degree of gonadal development and is defined as the ratio of gonad wet weight to the total sea urchin wet weight. Although Ebert and colleagues [29] report that serious errors may result from comparing GSI across sites or times without accounting for size of the individual, fishermen collect a very small size range of sea urchins. Nevertheless, we examined the relationship between test diameter and GSI.

Since two months did not include port-sampled red sea urchins from all three islands, a single model testing differences across all months and islands was not possible. Therefore, we assessed differences in GSI among islands for the two months with missing data (January and November) as a separate analysis and corrected for multiple tests using false discovery rate (FDR) adjusted p-values. For both ANOVAs, we sequentially removed non-significant interaction terms (above *P* > 0.05). Finally, we fitted a polynomial regression to each island in order to describe the functional forms of seasonal variation in GSI across islands.

### Red sea urchin industry quality and price

On average we sampled from nine boats per month (*n* = 40 unique boats total; on average 24 boats per year). To compare mean GSI per boat sampled per month (*n* = 258 sampling events) with the industry’s measure of sea urchin quality, we obtained data from the processor that included: date landed, price (USD) paid to the diver per load, weight of the highest quality sea urchin gonads (grade A and grade B uni; see S1 Appendix for more details on processor grading system and patterns), and weight of total load (whole sea urchins weighed at the dock). Using these data, we calculated the *processor quality index* (PQI) per fishing trip:

PQI=(gradeAtotalgonadweight+gradeBtotalgonadweight)/totalloadweight. (1)

The PQI is commonly referred to as *yield* or *gonad yield* or *recovery factor* within the sea urchin fishing industry [23] and indicates the fraction of high valuable product extracted from the entire catch. We requested data from seven processor companies who buy urchins from the Port of Santa Barbara. Only one processor was willing to provide data. We asked 18 divers for permission to use their fishing data from the single processor. Ten divers agreed to the use of their data, and these divers were responsible for providing 53% of our port samples.

Since price can fluctuate based on supply and demand of both domestic and international markets, we evaluated if local quality predicts price of red sea urchins despite temporal fluctuations in prices driven by global variation in supply and demand. We used linear regression to predict monthly mean price per kilogram from the monthly mean PQI. In addition, we tested whether the seasonal variability in red sea urchin gonads relates to processor perceptions of gonad quality. We tested how well the red sea urchin reproductive cycle (monthly mean port-sampling GSI) predicts the quality of sea urchin uni (monthly mean PQI) using linear regression.

### Red sea urchin fishing patterns

To examine our second objective, we investigated patterns in fishing behavior. To this end, we compared effort and landings during the period of limited fishing (four days per week during June-October) and the period with unlimited fishing (November-May). We also assessed seasonal differences in red sea urchin price and total value. Historical effort data showed that the western region was more heavily fished than the eastern region [35], and we suspected this was largely due to geographic differences in roe quality and value. The western region has higher densities of larger harvestable urchins with greater reproductive biomass potentially due to colder temperatures and more food availability (kelp) [36].

#### Comparing fishing metrics between regulatory time periods

To examine potential differences in fishing behavior across regulatory time periods, we examined total effort and total landings. The CDFW requires commercial sea urchin fishermen to submit a landing receipt for each trip, which contains information including fishing location and weight of the entire catch landed at the dock before sea urchins are processed. Total effort was measured as the sum of landing receipts submitted to CDFW per month. Since there are no comprehensive data on hours spent diving per trip, CDFW often uses the number of receipts as a proxy for effort. Since divers occasionally report multi-day trips on one landing receipt, this estimate of effort is likely an underestimate of total days of fishing. Total landings were the sum of landings (in kilograms) reported to CDFW per month.

In order to compare fishing effort during the limited versus the unlimited fishing time periods, we examined statistical differences (using ANOVA) in CDFW monthly total effort and total landings during 2009–2011 within the Channel Islands between the two management periods (limited: June-October and unlimited: November-May). We also examined CDFW data from the port of Fort Bragg to assess whether there were fishing behavior differences across management time periods within the region where the limited-fishing season was initially based.

#### Regional and seasonal fishing patterns

To examine regional and seasonal fishing patterns, we examined four fishing metrics (total effort, total landings, mean price, and total value) per month during 2009–2011 within four subregions (*west*, *central*, *east*, and *Anacapa*) of the Channel Islands as recorded in the CDFW landing receipts, using 10 x 10 minute numbered blocks (Fig 1). All subregions included four CDFW blocks, except Anacapa, which only included two blocks.

Effort and landings are explained in the section above. Mean price was the average price per kilogram (USD) of landed red sea urchins reported to CDFW per month. Total value was calculated as the monthly sum (USD) paid to all sea urchin fishermen as recorded by CDFW.

To evaluate regional variability in the red sea urchin commercial fishing data, we performed a series of ANOVAs, using the four monthly fisheries metrics across subregions. We corrected for multiple tests using FDR adjusted p-values.

Finally, to evaluate fishing effort and landings across regions during the times of the peak and trough of the red sea urchin reproductive cycle, we performed ANOVAs across the subregions during the months when GSI is lowest (April through June) and highest (September through December). We included region, season, and the region by season interaction to predict effort and landings.

### Relating commercial fishing data to red sea urchin reproduction

Our third objective was to investigate how fishermen respond to variation in reproductive condition of red sea urchins. We explored patterns in fishing behavior, including whether fishermen on average harvest more in peak quality seasons and locations to garner better prices. We tested whether the industry’s effort, total landings, and value were correlated with the seasonal patterns in the demographic measure of GSI. If global prices vary widely, local quality in the product may play a minor role in determining prices. Conversely, if variation in urchin prices is driven mostly by urchin quality, as measured by GSI, rather than global fluctuations in supply and demand, seasonal and geographical patterns of GSI could give insight into both sea urchin demographics and the resulting behavior of fishermen.

We tested whether the seasonal reproductive stage, measured as GSI, was a good predictor of fishing behavior (effort and landings), red sea urchin price, and value using CDFW metrics. We used the fisheries data across the same years of our GSI samples (2009–2011) and across all available data from previous years of the fishery (1978–2008) with the rationale that seasonal fishing behavior was likely to be driven by knowledge of red sea urchin reproductive dynamics and the assumption that the red sea urchin’s reproductive cycle has remained relatively consistent over the years. We have evidence from a one-year study performed in the Channel Islands in 1975–1976 that GSI had a similar cycle [37], and we wanted to examine whether seasonal variability in industry metrics has remained consistent over the years.

Since the majority (72%) of our port sampling came from the western four blocks within the northern Channel Islands, we had more consistent monthly data from this area. We compared the average monthly GSI from these western samples to the monthly total effort, total landings, mean price, and total value data from these same locations using linear regression.

## Results

### Red sea urchin seasonal reproductive cycle

**Fig 2:**
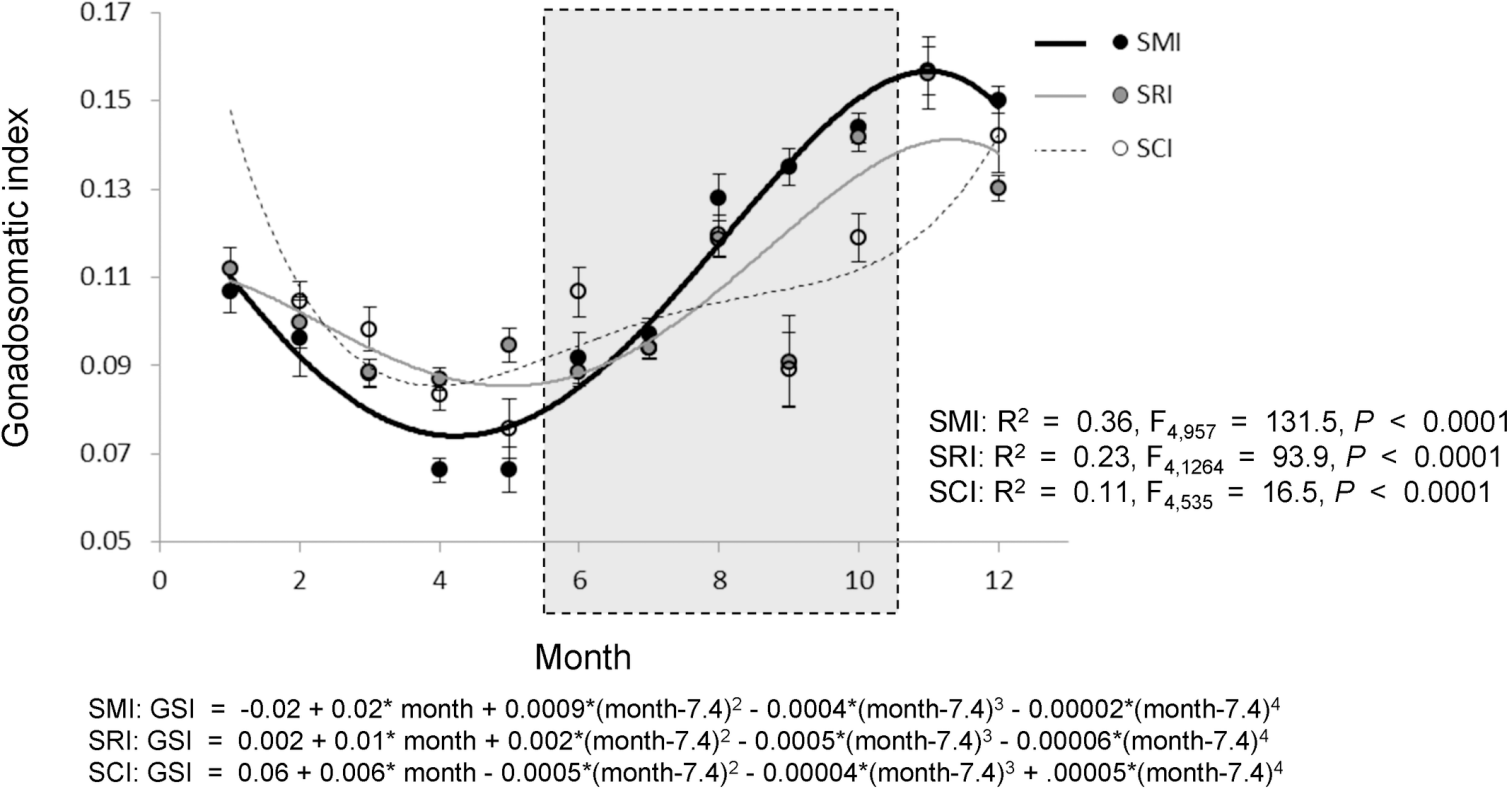
Red sea urchin seasonal reproductive cycle. Monthly mean gonadosomatic index per boat sampled from red sea urchins landed at the port of Santa Barbara from December 2008 to December 2011 per island: San Miguel Island (SMI), Santa Rosa Island (SRI), and Santa Cruz Island (SCI); error bars show one standard error. Lines show the quartic polynomial fits for viewing purposes only of the monthly means per island. The gray box highlights the months when fishing is limited to four-days per week.

Gonadosomatic indices varied among regions in a marked, but complex, seasonal pattern (ANOVA: F_30,2496_ = 34.7, *P* < 0.0001, R^2^ = 0.30; Fig 2, S2 Appendix). The quadratic polynomial regressions characterized the seasonal changes in red sea urchin GSI and how GSI varied from island to island (Fig 2; all *P-*values < 0.0001). The reproductive cycles of red sea urchins within the three islands have similar phases but appear to have different amplitudes. Red sea urchin GSI was greatest in the fall (November GSI = 0.157 ± 0.005), where it was almost double the indices we observed in the spring months (April and May GSIs were 0.080 ± 0.002 and 0.085 ± 0.003, respectively). When we examined the 10 months of GSI means across the three islands, GSI differences among the islands were greatest during the two extreme periods of the reproductive cycle–the peak of GSI (September through December) and the trough of GSI (April and May) (Fig 2, S2 Appendix). The GSI gradient among islands reversed directions during these periods. At its peak, GSI decreased from west to east (by about 13% in December), while at the trough of the reproductive cycle, GSI increased from west to east (by about 32% in April). The GSI of sea urchins from the easternmost island (SCI) was the least variable across seasons. Within the two months (January and November) when we had samples only from SMI and SRI, there were no differences between the two islands, but GSI in November was significantly higher than in January (ANOVA: F_3,210_ = 22.3, *P* < 0.0001, R^2^ = 0.24; S2 Appendix).

Test diameter on average was 97 mm at SCI, 101 mm at SRI, and 101 mm at SMI and varied within each island across months only by ± 0.9, 0.8, and 1.3 mm standard error of the mean, respectively. In addition, there was a very weak, significant bivariate relationship between GSI and test diameter (linear regression; R^2^ = 0.00018, *P* = 0.0248; slope = 0.00021; y-int = 0.09; N = 2752). Given the extremely small R-squared value, small positive slope, and high sample size, perhaps this result is not ecologically significant. Thus, for ease of presentation and to be comparable across numerous published work on GSI, we did not account for these small differences in size.

### Red sea urchin industry quality and price

**Fig 3:**
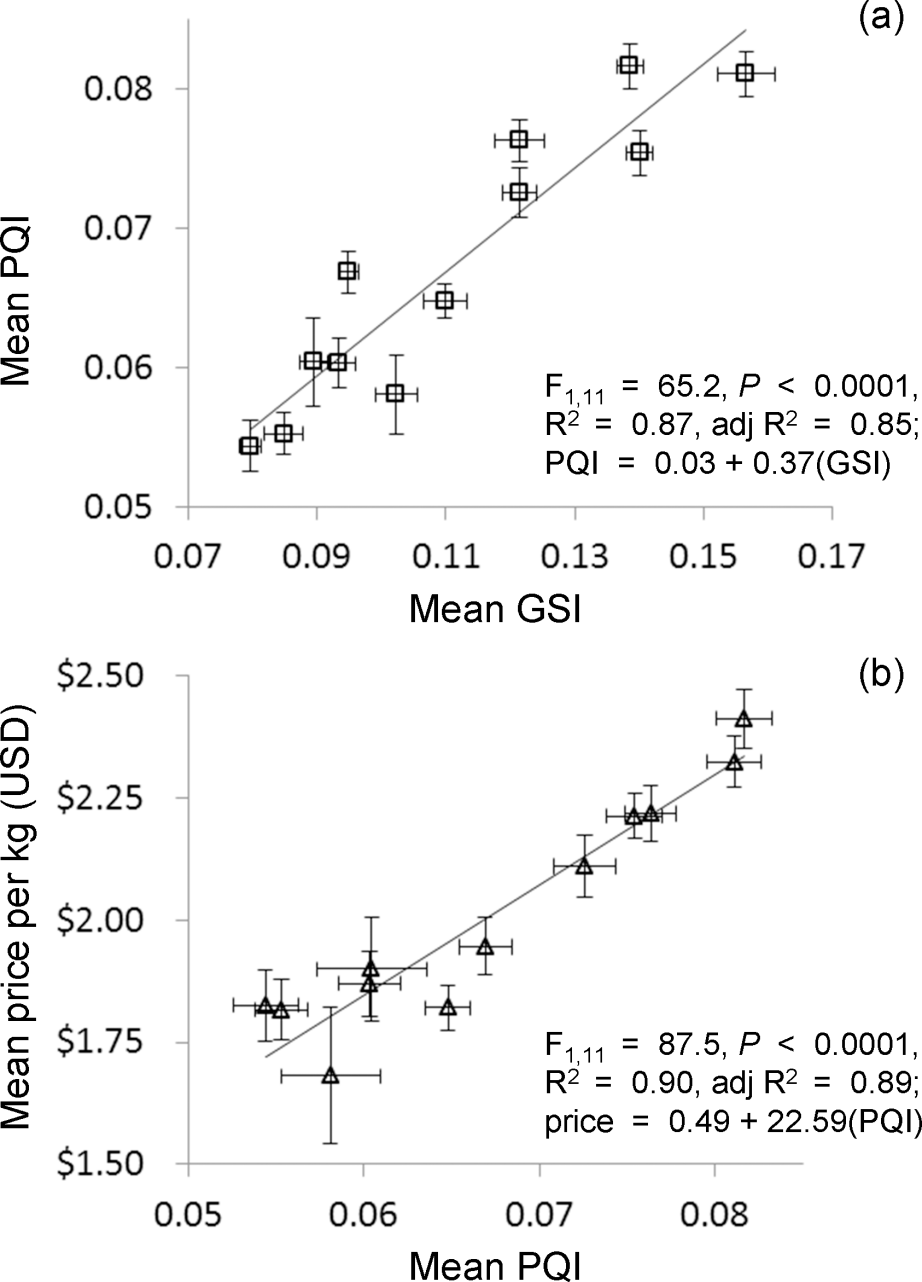
Processor quality index, price, and gonadosomatic index. Processor data regressions: (a) mean port-sampling gonadosomatic index (GSI) predicting mean processor quality index (PQI) and (b) mean PQI predicting mean processor price per kg (USD). Error bars show one standard error.

The reproductive cycle of red sea urchins, processor gonad quality, and price were tightly correlated. Monthly mean red sea urchin GSI was a significant positive predictor of our processor quality index (PQI) (linear regression; R^2^ = 0.87, β_1_ = 0.93, *P* < 0.0001; Fig 3A). In addition, PQI was a strong positive predictor of mean processor price per kg (USD) (linear regression; R^2^ = 0.90, β_1_ = 0.95, *P* < 0.0001; Fig 3B). On average per month, it appears that price is largely determined by quality rather than fluctuations in global market drivers.

### Red sea urchin fishing patterns

#### Comparing fishing metrics between regulatory time periods

**Fig 4:**
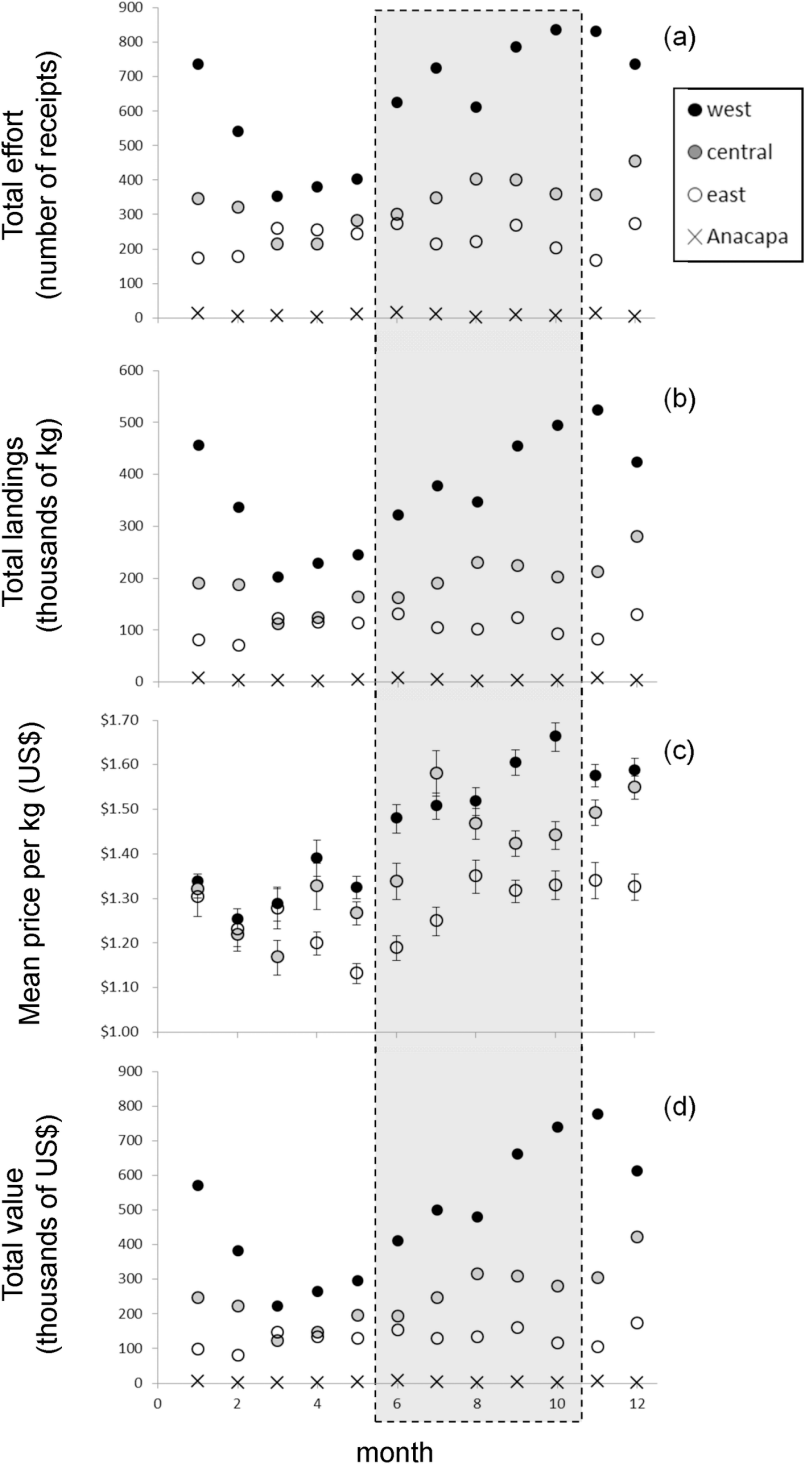
Red sea urchin monthly commercial fishing. Monthly data from California Department of Fish and Wildlife (CDFW) in commercial red sea urchin (a) total effort (number of receipts), (b) total landings (thousands of kg), (c) mean price per kg ± one SE (USD), and (d) total value (thousands of USD) for the northern Channel Islands fishery per year (2009–2011) within the four subregions (see Fig 1). (Note: Anacapa was excluded from plot (c) mean price per kg due to extreme outliers and since less than 1% of the receipts, landings, and value came from Anacapa.) The gray box highlights the months when fishing is limited to four-days per week. Summary statistics are provided in Table 1.

Monthly total effort and total landings did not differ significantly between the limited and unlimited fishing management seasons (Two-way ANOVA; model = effort: F_1,23_ = 1.38, *P* = 0.25; model = total landings: F_1,23_ = 0.48, *P* = 0.49; Fig 4; Table 1). Both fishing effort and total landings were at least 65% greater at the Channel Islands compared to the Fort Bragg region (Two-way ANOVA; model = effort: F_1,23_ = 143.82, *P* < 0.001; model = total landings: F_1,23_ = 99.08, *P* < 0.001; Table 1). We saw some evidence that fishing effort varied between management seasons at the Channel Islands, although this result was not statistically significant in our model (Two-way ANOVA; season x region: F_1,23_ = 3.66, *P* = 0.07; Fig 4). Within Fort Bragg there were clearly no differences in either metric between seasons, but within the Channel Islands there was a slight trend of greater total effort (number of receipts) during those months limited to a four-day work-week compared to the unlimited period. On average a vessel submitted 3.6 ± 0.15 SE receipts per week during the limited management season and 3.2 ± 0.14 SE receipts per week during the unlimited management season.

**Table 1:** Analysis of variance in red sea urchin fishing effort and landings. (a) Mean total effort (number of receipts) and total landings (thousands of kg) during 2009–2011 per management season (months with 4-day work weeks [*limited* access] and months with no management restrictions [*unlimited* access]) and region (14 CDFW blocks within the Channel Islands [CI] and the port of Fort Bragg [FB]), and one standard error (SE) are displayed. (b) Two-way ANOVA results testing the differences in commercial red sea urchin total effort and total landings, between management (mgmt.) seasons, and between the two regions.

**(a)**									
**region**		**total effort**				**total landings**			
		**limited**		**unlimited**		**limited**		**unlimited**	
		**(June-Oct)**		**(Nov-May)**		**(June-Oct)**		**(Nov-May)**	
		**mean**	**SE**	**mean**	**SE**	**mean**	**SE**	**mean**	**SE**
**CI**		1325	94	1113	79	710	62	628	53
**FB**		367	47	418	40	221	26	242	22
**(b)**									
		**total effort**				**total landings**			
	**DF**	**SS**	**MS**	**F Ratio**	***P***	**SS**	**MS**	**F Ratio**	***P***
**model**	3	4,022,157	1,340,719	48.4	< 0.0001	1.10E+12	3.80E+11	33.3	< 0.0001
**mgmt. season**	1	38,286	38,286	1.4	0.2535	5.40E+09	5.40E+09	0.5	0.4979
**region**	1	3,983,661	3,983,661	143.8	< 0.0001	1.10E+12	1.10E+12	99.1	< 0.0001
**mgmt. season x region**	1	101,354	101,354	3.7	0.0702	1.60E+10	1.60E+10	1.4	0.2537
**residual**	20	553,978	27,699			2.30E+11	1.10E+10		
**total**	23	4,576,135	198,962		**R**^**2**^ **= 0.88**	1.40E+12	5.90E+10		**R**^**2**^ **= 0.83**

#### Regional and seasonal fishing patterns

Although there were no significant differences among metrics between the two regulatory time periods, we found significant regional and seasonal variability in the fishery metrics (monthly total effort, total landings, mean price, and total value) during 2009–2011 (Fig 4, Table 2). Among the three westernmost subregions, the most heavily fished west subregion, showed the greatest seasonal variation in monthly total effort, total landings, and total value (based on the higher CVs, Table 2A). Levels of these three fishery metrics generally were lower in the spring and higher in the fall and winter (Fig 4). Mean prices of red sea urchins from the two western subregions showed a similar magnitude of intra-annual variability (the CVs were comparable, Table 2A). In addition, within all three westernmost subregions, mean prices generally increased from February through the end of the year (Fig 4C). Since total effort, landings, and value from Anacapa were very low, we excluded this subregion from further analyses. Within the Channel Islands region, all fishery metrics decreased from west to east (One-way ANOVAs; Table 2). Total effort, landings, and value in the west were on average about 48% higher than in the central subregion and about 70% higher than in the east subregion. On average prices within the west and central subregions tended to be 11% higher than in the east subregion. False discovery rate post-hoc analyses p-values for these four tests were below the 0.05 threshold.

**Table 2:** Regional variation in red sea urchin fishing. Regional variation in commercial fishing data (CDFW) from the Channel Islands (2009–2011): total effort (number of receipts), total landings (kg), mean price per kg (USD), and total value (USD). (a) seasonal variation summary statistics of monthly (n = 12) data: mean, standard error (SE), and coefficient of variation (CV), and (b) Regional variation among the three western subregions within the Channel Islands (Fig 1) ANOVA results and post hoc Student’s t-test. Levels not connected by the same letter are significantly different.

**(a)**												
	**west**			**central**			**east**			**Anacapa**		
	**mean**	**SE**	**CV**	**mean**	**SE**	**CV**	**mean**	**SE**	**CV**	**mean**	**SE**	**CV**
**total effort**	631	51	27.8	334	21	21.7	228	12	17.6	8	1	57.6
**total landings**	366,464	30,758	29.1	188,801	13,360	24.5	104,634	5,810	19.2	2,626	584	77.0
**mean price**	$1.46	$0.04	9.4	$1.38	$0.04	9.4	$1.27	$0.02	5.5	$2.53	$0.54	74.2
**total value**	$493,517	$53,143	37.3	$251,213	$23,823	32.9	$130,497	$7,801	20.7	$3,158	$556	61.0
**(b)**												
**ANOVA results**										**Post hoc Student's t-test**		
		**DF**	**SS**		**MS**		**F Ratio**	***P***	**R**^**2**^	**west**	**central**	**east**
**total effort**	**region**	2	1,042,797		521,399		41.6	< 0.0001	0.72	A	B	C
	**residual**	33	413,245		12,523							
	**total**	35	1,456,042									
**total landings**	**region**	2	4.29E+11		2.14E+11		46.3	< 0.0001	0.74	A	B	C
	**residual**	33	1.53E+11		4.63E+09							
	**total**	35	5.82E+11									
**mean price**	**region**	2	0.22		0.11		8.1	0.0014	0.33	A	A	B
	**residual**	33	0.45		0.0136							
	**total**	35	0.67									
**total value**	**region**	2	8.20E+11		4.10E+11		29.7	< 0.0001	0.64	A	B	C
	**residual**	33	4.56E+11		1.38E+10							
	**total**	35	1.28E+12									

Finally, when we evaluated regional fishing effort and landings during the times of the peak red sea urchin reproductive cycle (the months of September through December), we found significant differences across the three subregions (Two-way ANOVA region x GSI season; model = effort: F_2,20_ = 13.3, *P* = 0.0005; model = total landings: F_2,20_ = 18.5, *P* < 0.0001; Table 3). Regional patterns in effort and landings during the months of peak GSI were similar to the average annual differences, with the west on average 51% higher than the central subregion, and 74% higher than the east subregion (Table 3). However, during the months when GSI is lowest (April through June), the three subregions were more similar in effort, landings, and price. Despite this, the west subregion had effort and landings that were higher than the other subregions, but the central and east subregions were not significantly different and were on average 69% lower than the west subregion.

**Table 3:** Regional and seasonal variation in red sea urchin fishing. Regional and seasonal variation in commercial fishing effort and landings (CDFW) from the three western subregions within the Channel Islands (2009–2011): total effort (number of receipts) and total landings (kg) across two red sea urchin gonadosomatic index (GSI) seasons (months of *lowest* [trough: April through June] and *highest* [peak: September through December]). (a) Two-way ANOVA results and (b) post hoc Student’s t-tests. Levels not connected by the same letter are significantly different. Least square (LS) means and standard error (SE) are displayed. Highest GSI season per region are highlighted in gray for ease of comparison.

**(a)**										
		**total effort**				**total landings**				
	**DF**	**SS**	**MS**	**F Ratio**	***P***	**SS**	**MS**	**F Ratio**		***P***
**model**	5	875,101	175,020	42.4	< 0.0001	3.60E+11	7.20E+10	62.4		< 0.0001
**region**	2	576,855	288,427	69.9	< 0.0001	2.40E+11	1.20E+11	103.2		< 0.0001
**GSI season**	1	103,376	103,376	25.0	0.0002	4.40E+10	4.40E+10	38.2		< 0.0001
**region x GSI season**	2	109,416	54,708	13.3	0.0005	4.20E+10	2.10E+10	18.5		< 0.0001
**residual**	15	61,930	4129			1.70E+10	1.20E+09			
**total**	20	937,031			**R**^**2**^ **= 0.93**	3.80E+11				**R**^**2**^ **= 0.95**
**(b)**										
**region, GSI season**				**total effort**		**total landings**				
				**LS Mean**	**SE**	**LS Mean**	**SE**			
**west, highest**				798	32	473,155	16,936	A		
**west, lowest**				470	37	263,765	19,556		B	
**central, highest**				394	32	228,808	16,936		B	
**central, lowest**				266	37	148,999	19,556			C
**east, lowest**				258	37	118,579	19,556			C
**east, highest**				229	32	106,365	16,936			C

### Relating commercial fishing data to red sea urchin reproduction

**Fig 5:**
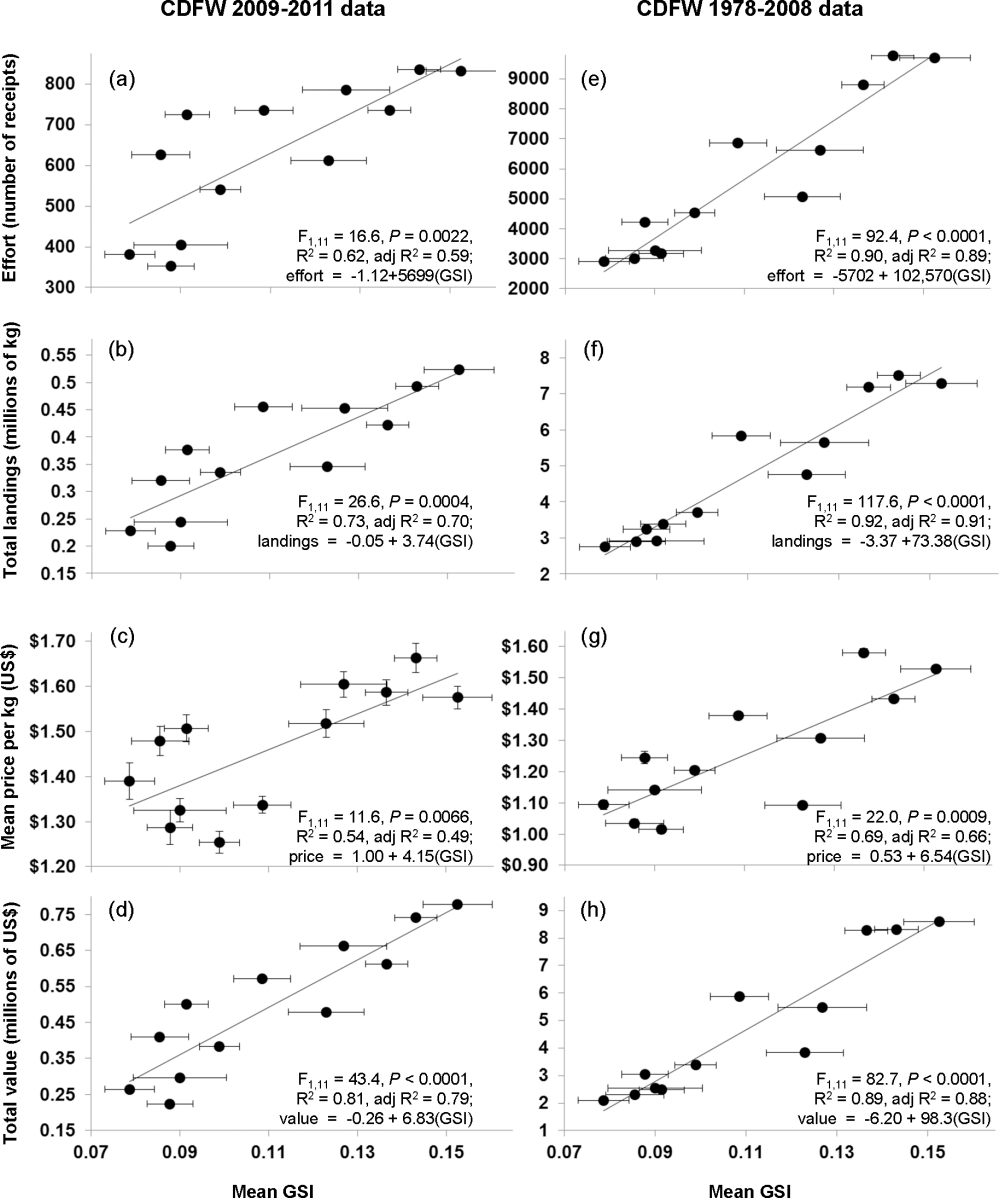
Predicting red sea urchin fishing from gonadosomatic index. Mean port-sampling gonadosomatic index (GSI) predicting average monthly California Department of Fish and Wildlife (CDFW) data for 2009–2011 and for 1978–2008 in (a, e) effort (number of receipts), (b, f) total landings (millions of kg), (c, g) mean price per kg (USD), (d, h) total value (millions of USD) within the *west* subregion (see Fig 1). Error bars show one standard error (note: many error bars are smaller than marker size).

Not surprisingly, within the most heavily fished subregion (the west) on a monthly basis, sea urchin fishermen predictably harvest red sea urchins according to their reproductive cycle. As red sea urchin gonadosomatic index (GSI) increased, monthly total effort (linear regression; β_1_ = 0.79), total landings (linear regression; β_1_ = 0.85), mean price (linear regression; β_1_ = 0.73), and total value (linear regression; β_1_ = 0.90) significantly increased during 2009–2011 (Fig 5). These patterns were mirrored in the historical time period (1978–2008); as GSI increased, monthly total effort (linear regression; β_1_ = 0.95), total landings (linear regression; β_1_ = 0.96), mean price (linear regression; β_1_ = 0.83), and total value (linear regression; β_1_ = 0.94) significantly increased. When red sea urchin gonad condition was greatest, fisherman on average expended greater effort and produced larger landings, which is consistent with the higher prices paid to fishermen during the peak season. Conversely, when gonad quality was poorer, fishermen tended to fish less for urchins, and they received lower prices.

## Discussion

We observed spatial and temporal differences in red sea urchin reproduction, which explained much of the seasonal and spatial variation in fishing effort, landings, and urchin value. Red sea urchins exhibited a pronounced annual reproductive cycle in the northern Channel Islands that differed substantially from published patterns in northern California [27]. However, statewide fishing regulations were developed based on seasonal dynamics in northern California. A better understanding of the linkages between sea urchin reproduction and fishing behavior and how this may vary across regions could help managers make more effective decisions.

### Red sea urchin reproduction and quality

No previous studies have described in detail the entire annual reproductive cycle of the red sea urchins in the heavily fished region of the Channel Islands. Other published information on GSI for red sea urchins was limited in geographic range (within Oregon, Los Angeles area, San Diego area, and within Baja California, Mexico; [38]), years, and season (number of months per year examined). For several of the ranges examined there was one full annual cycle studied, but we have not found any previous studies examining multiple annual cycles within a particular range.

Our results show the spawning period in the northern Channel Islands occurred over roughly five months (December-April), and the building, or gonad growth, period occurred over roughly seven months (May-November) (Fig 2). The seasonal patterns we found in the reproductive cycle (GSI) and processor gonad-yields (PQI) generally match those reported in a one-year study that took place in the early years of the fishery (1970’s) of sea urchins from San Miguel and Santa Cruz Islands and a one-year study of processed sea urchins from southern California [37]. In addition, Ebert and colleagues [39] reviewed literature reporting a similar winter to spring timing of spawning for red sea urchins in southern California. Spawning in northern California was noted to be later, occurring in the spring to summer seasons [39]. Furthermore, previous research on the co-occurring purple sea urchin *Strongylocentrotus purpuratus* reports a similar annual cycle [40–42].

Finally, we saw an interesting pattern with GSI showing a lower amplitude cycle for sea urchins from eastern (warmer) locations. Previous research has indicated that purple sea urchins may show a similar pattern of lower amplitude cycles in warmer locations (Baja California in comparison to California and south in comparison to north of Point Conception) [41,43].

### Potential drivers of red sea urchin reproduction and quality

As in many species, these seasonal patterns of reproduction may be driven by seasonal patterns in resource abundance or quality for adults or larval stages [32,34,44–46]. Previous studies have shown that food availability and quality for adult sea urchins influences gonad quality [47–49]. In the spring months, kelp begins to recover from winter storm disturbance [50], which is synchronous with the increase in red sea urchin allocation to reproductive growth. There is high inter-annual variability in kelp canopy biomass, but it generally peaks at SMI, SRI and SCI around June through August [50,51] during the period of peak GSI increase. In addition, drift kelp, an important resource for sea urchins, tends to be higher in the summer and fall, when kelp biomass is higher and water movement is lower [41,43,52,53]. Following the timing of high abundance of drift kelp, purple sea urchins have shown subsequent peaks in both the stomach (percent weight of stomach, intestines, and gut contents of the total body weight) and gonad indices [54]. Within our study region, kelp canopy biomass is generally lowest in the winter months due to age-dependent mortality [55] and disturbance in response to increased wave heights from winter storms [50,56,57]. The spawning period of red sea urchins coincides with the period of minimum kelp biomass. Thus, spawning occurs in the months when resources for adults are more limited, so there is less opportunity to garner new resources to support gonadal growth.

As with other urchin species, if food is limiting to larval success, we would also expect spawning to coincide with phytoplankton blooms (the primary resource for the larval stages of sea urchin), rather than temperature [46,58–60]. Sea urchin larvae begin to feed within the first week of life and remain in the water column for about 40 days (ranging from 27 to 131 days depending on food and temperature; [61,62]). Recent data (1997–2015) suggest that the Santa Barbara Channel experiences extreme inter-annual variability in the timing of chlorophyll peaks, but in general blooms begin between March and June, with some years starting in February and some peaking in September [63,64]. However, red sea urchin spawning begins and peaks in December and January and appears to continue through June (Table A in S3 Appendix). While the timing of peaks in phytoplankton and red sea urchin spawning do not appear to be perfectly aligned, the month of lowest levels of chlorophyll and highest sea surface temperatures in September coincides with the lowest spawning levels observed in this study (Table A in S3 Appendix; [63]). The reproductive timing of red sea urchins is likely tightly linked with both adult resources (kelp) and larval resources (phytoplankton) [65]. However, further studies are needed to disentangle the relative influence of temperature and food availability (specific to various life-cycle stages) on the reproductive timing of sea urchins, and there are a number of other factors to consider (e.g., currents, topography, settlement timing, habitat quality).

### Fishermen respond to the reproductive timing of the fished species

It may be common knowledge that fishermen respond to the demographic timing of the fished species, in particular reproductive timing. However, we have quantified how a simple reproductive metric (GSI) can be used to predict industry metrics, which can in turn be useful for management strategy evaluation and planning.

Fishermen respond to red sea urchin reproductive variability due to differences in roe quality and price. During, the beginning of the spawning season (November through December) prices are still relatively high but then they drop rapidly as spawning continues (January through April). Our results indicate that red sea urchins are more valuable in the western channel, especially during peak GSI in the fall. Consistent with this pattern, fishermen harvest more in western locations than eastern locations, especially during this period (Fig 4; Table 3; [35]). By contrast, during the trough of the reproductive season, GSI showed the opposite spatial pattern–lower in the west than in the east (Fig 2 and Figure A in S2 Appendix). During the trough of the reproductive season, fishermen still fished more in the west subregion, but the regional differences in fishing effort and landings were not nearly as pronounced during this time of year (Table 3). There were no significant differences in effort and landings between the central and east subregions (Table 3). Fishermen likely do not more aggressively switch to harvesting more sea urchins in the east during the period of low GSI because of the higher abundance of larger and potentially more valuable urchins in the western regions [36]. In addition, an interesting pattern surfaced opposing our prediction that effort would steadily increase tracking the gonad growth after the annual low point (in April); by June effort in the western region increased higher than expected and this happens to be the month when effort restrictions are in place. We suspect this pattern is partially due to the more favorable summer weather after spring winds have subsided allowing for a greater number of days fished—especially in this more exposed western region. Also, during this time the gonad quality is high, so fishermen likely are “racing” to collect as many urchins as possible before their fellow fishermen do, especially since in recent years this period coincides with heavy domestic demand (D. Rudie, pers. obs.).

When we examined the most heavily fished subregion, the west, we found that high temporal variation in the quality of a fished resource drove predictable seasonal patterns of fishing. We found that quality, total effort, total landings, mean price, and total value in sea urchins harvested from the Channel Islands are highly predictable based on the reproductive cycle, measured here as GSI. These fishery metrics and GSI during our sampling period (2009–2011) were significantly and strongly related (Fig 5). In addition, we used these GSI data to predict historical metrics of the fishery (1978–2008). These relationships were similar and stronger indicating that the red sea urchin’s reproductive cycle has been a strong driver of the sea urchin industry patterns over those 30 years of the fishery. Our results from southern California corresponded with historical data from northern California showing gonad yield and price to be positively correlated (Figs 3 and 5; [27]). However, historically the catch in northern California was inversely related to price (data 1985–1994; [27]), which was contrary to our findings. Fishermen may have been somewhat limited by unsafe boating conditions in the winter, when prices tended to be higher in northern California [27], and because of these constraints they fished more during the season of low prices.

### Implications

**Fig 6:**
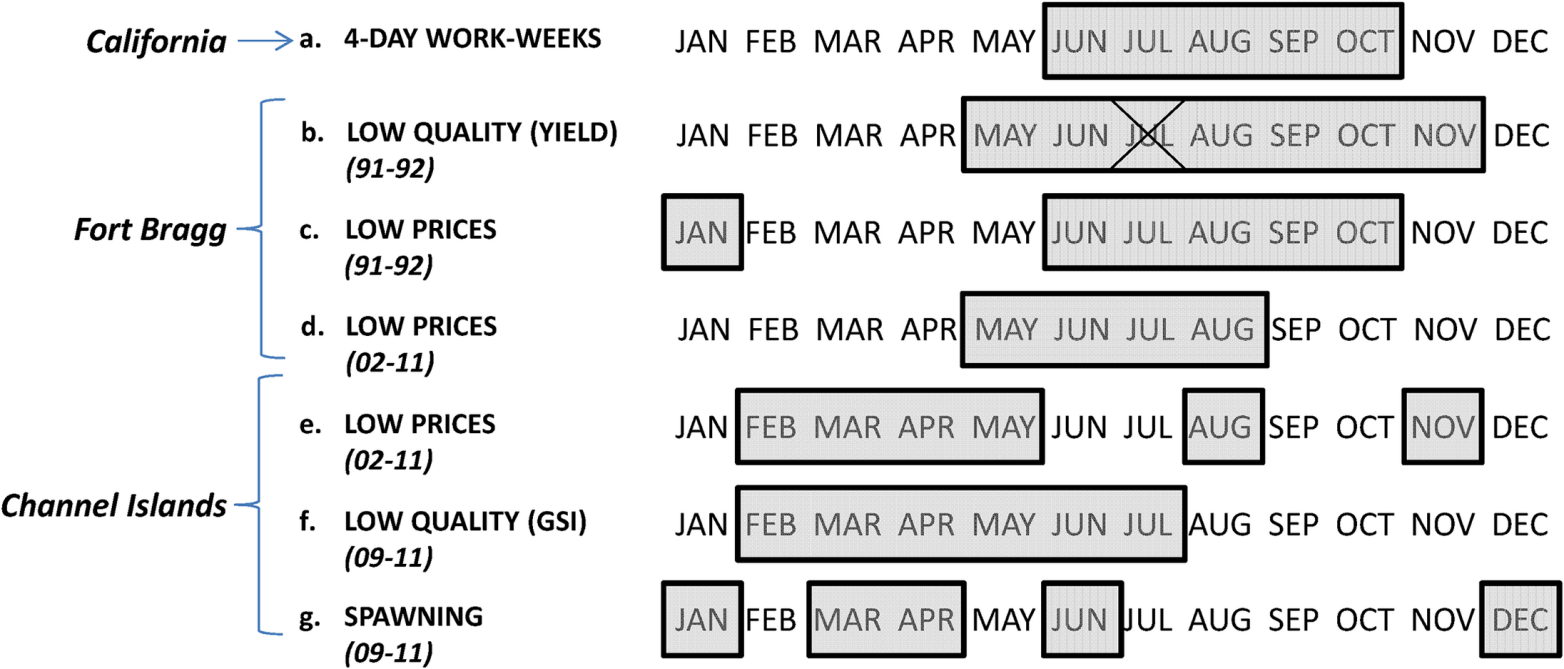
Regional comparison of the california sea urchin fishery. Comparing months across the California sea urchin fishery within Fort Bragg in northern California and the Channel Islands in southern California. Gray highlights: (a) the five months with limited fishing across the state of California (commercial sea urchin fishermen are allowed to fish four-days per week during these months); the rest of the year there is unlimited fishing; (b) the six months with the lowest quality (yield) from a processor in Fort Bragg 1991–1992 (Table B in S3 Appendix), the location and time-frame which was examined to establish the four-day work weeks; July is excluded due to the fishery being closed during this period; (c) the six months with the lowest prices in Fort Bragg 1991–1992 (Table C in S3 Appendix); (d) the four months with the lowest prices in Fort Bragg 2002–2011 (Table D in S3 Appendix), (e) the six months with the lowest prices in the Channel Islands 2002–2011 (Table E in S3 Appendix); (f) the six months with the lowest quality gonadosomatic index (GSI) in the Channel Islands 2009–2011 (Table B in S2 Appendix) and (g) the five months with the highest spawning levels in the Channel Islands 2009-2011(Table A in S3 Appendix).

Management restrictions were established in California in order to limit harvest during the low gonad quality season with the rationale that if effort needs to be limited to regulate overall catch, the costs would be lower if sea urchin fishermen had greater effort during a season of higher quality. This statewide management scheme was based on the cycle of the northern California red sea urchin; managers attempted to limit effort during the season with low quality and prices (in the late spring to fall months) (Fig 6). Currently, fewer work-days are allowed statewide during the months of June through October, but in southern California this is the middle through nearly the end of the gonadal growth period (Figs 2 and 6). These months of restricted fishing include several months when fishermen received some of the highest prices (Fig 6 and Table E in S3 Appendix). We have identified a mismatch in management goals and outcomes due to the variability in the demographics of this species across its management range, which is a common occurrence in ocean governance worldwide [66–71].

Our results show that the period of low prices, low quality, and high spawning at the Channel Islands (winter to spring) generally did not coincide with the period during which managers attempt to curtail effort through limiting allowable days fishing (summer to fall). The time of the year with a limited number of allowable fishing days did not result in significantly lower monthly effort (number of days fished) than the rest of the year (Table 1). If anything, there was a weak trend that effort and prices were lower within the Channel Islands during the unlimited season, which reflects the fact that this unlimited time-period contained the months with the lowest GSI (Fig 2). Part of the unlimited time-period also coincides with more frequent storms and high wind speeds of winter and spring [72,73], which may also limit fishing trips statewide. As with other open access fisheries, there is little incentive to conserve or limit harvest [74], especially during a highly-profitable season. The state of California Fish and Game Commission (CFGC) has considered introducing a total allowable catch but the cost of enforcing such harvest restrictions was determined to be too costly [75]. The CFGC is currently adopting the regulation change of reducing capacity from 300 to 150 permits, which is important to reduce latent capacity to protect the industry from unexpected spikes in demand and effort. However, this action may not reduce effort in the near future since 150 divers (2007–2016) harvest 97% to nearly 100% of the landings.

Although California does not close its fishery during the spawning season, the current fishing-day restrictions in southern California, may in fact be providing a conservation benefit to the fishery. Management is restricting fishing days during part of the period of high quality urchin gonads, when the fishermen would want to increase effort, during the middle of the gonad building period. Fishermen are fishing close to the maximum days allowed during this time period (submitting 3.6 ± 0.15 receipts per week on average per boat) indicating that regulations are likely limiting effort. Although we did not detect a difference in effort between the two management time-periods, this scheme may be effectively curtailing effort. If no fishing-day restrictions were in place, they could theoretically fish 40% more per week (three more fishing days). In 2014, the California Sea Urchin Commission, sea urchin processors, and buyers specifically requested adding one more open day per week to the summer to early fall months, when demand tends to be high in the US market in recent years [76] to enhance the profitability of the fishery. In response, the California Fish and Game Commission decided to allow fishing during one additional day per week (Friday) for June to October in Southern California only (south of the Monterey-San Luis Obispo county line). This regulation change was adopted at the end of 2017 and will be effective by spring of 2018 (S. Ashcraft and S. Tiemann, pers. comm.; [75]). However, it is unknown whether the resource could sustain up to 14% more intense fishing (by adding one more day per week) during this season, which occurs right before sea urchins spawn.

If managers seek to protect spawning biomass, they could consider two management actions. First, managers could restrict harvest during the spawning period, as many other fisheries around the world employ this management strategy. In southern California the fishery is unrestricted during the month of highest gonad quality and prices, when spawning begins, and is also unrestricted during the entire spawning period. Second, although current minimum size limits may be protecting the nursery stock of the population, implementation of a maximum size limit to protect the brood stock is also a traditional management approach [77] that may be useful for this fishery. Individual sea urchin size indicates the relative reproductive contribution to the population, since larger urchins (and fish species) have exponentially larger gonads and thus contribute a disproportionate amount of larval supply than sea urchins below the minimum size limit [77,78]. In addition, these larger sea urchins provide canopy shelter for recruits [37,79]. Furthermore, very large sea urchins are not marketable (Dave Rudie, pers. obs.); above 125 mm in test diameter quality declines [78], and the sushi industry relies on a uniform product of similar-sized uni [23]. Thus, only 1% of our port-sampled sea urchins were above 125 mm (and 3% were greater than 120 mm), however of sea urchins above the minimum size limit within a recent ecological survey in the same region 5% were greater than 125 mm (and 8% were greater than 120 mm) [36]. A slot size limit to protect these larger individuals may serve as a precautionary approach to management, as the State of Washington has also enacted [78], especially if market forces shift to preferring a larger product. In 1993, CDFW wrote a draft management plan, which included setting a maximum size limit among other measures such as implementing a total allowable catch, separating permitting process between northern and southern California, and closing the fishery statewide from June to September. The draft plan was extremely disfavored by the CDFW Director's Sea Urchin Advisory Committee and the industry [80], and likely today the sentiments would be similar.

A more adaptive management regime may be required in order to respond quickly to local population dynamics especially those associated with rapid ecological or environmental changes. Reserves can bolster ecosystem resilience to mass mortality events, climate change, and other stressors while supporting biodiversity and sustainable fisheries [81,82]. Recently (2011), marine protected areas (MPAs) in the region were reported to have a greater biomass of red sea urchins [36]. However, MPAs will not wholly safeguard resources from rapid and extreme changes such as warm water events, harmful algal blooms, increased storm frequency, and disease outbreaks. During 2015–2016 there was a widespread die off of sea urchins (up to 50%) due to wasting disease and black spot disease at all the Channel Islands and nearby mainland areas; localized outbreaks of these diseases continued in 2017 (D. Kushner and D. Reed, pers. comm.). In addition, the extreme warming event associated with the 2014 warm water blob and the 2015–2016 El Niño [83,84] may not have severely impacted kelp forests in all of southern California (at least initially in the Santa Barbara and Los Angeles areas) [85] but has been associated with widespread losses of kelp especially in San Diego (T. Bell, pers. comm.) and in some places up to 93% loss of *Nereocystis luetkeana* bull kelp forests in northern California (M. Carr, C. Catton, and L. Rogers-Bennett, pers. comm.; [86]). In northern California, the increase in purple sea urchin populations (by up to 60 times in some places) partially due to their lack of predators also appears to be a factor in the loss of kelp (M. Carr, C. Catton, and L. Rogers-Bennett, pers. comm.; [86]). One important predator to sea urchins the sunflower sea star *Pycnopodia helianthoides* suffered massive declines in the western Channel Islands and northern California due to another disease outbreak in 2013 (M. Carr, C. Catton, D. Kushner, and L. Rogers-Bennett, pers. comm.; [86–88]). These recent environmental and ecological changes have likely contributed to the 35% reduction in landings in southern California, an 80% reduction in landings in Northern California, and a 46% reduction in landings statewide (comparing 2006–2015 to 2016 CDFW annual landings data). In addition, the supply of high quality red sea urchin roe has declined (by about 50% in 2016–2017) likely due to a decline in food availability (D. Rudie, pers. obs.) and potentially disease. Diseased purple sea urchins have been reported to have lower gonad indices [89]. Although the fishery still has a minimum size limit and will further reduce the number of permits, loosening fishing-day restrictions could have unintended consequences for the future sustainability of the resource.

Our results, while underscoring the tight link between variability in a resource and fishing, are not embedded in a static world. We have quantified the extent to which reproductive cycles can drive seasonal quality in a resource, which in turn influences price and fishing effort. Future key research should include a consideration of how climate may influence both resource dynamics and fishing behavior. Changes in climate (e.g., increases in temperature, storm severity, and storm frequency) may result in both profound ecological ramifications [90–94] and varying fishing behavior [95]. For example, if storms increase during the fall, fishing effort during the high-quality gonad season may be more limited. If the higher frequency of storms and increased wave action reduces kelp density, gonad quality may be degraded in certain areas that were once important fishing grounds. In addition, since climate and fishing both influence species’ distribution and abundance, it is important to understand their combined effects on the system may be synergistic [96]. Examining phenological changes in species, which may include tracking reproduction over seasons and years, is not only important for resource management but also may be a simple ecological indicator of change [97].

## Supporting information

S1 Appendix Sea urchin processor quality evaluation and seasonal data. (PDF)

S2 Appendix Red sea urchin gonadosomatic index: Temporal and spatial details. (PDF)

S3 Appendix Monthly variation of red sea urchins: Spawning and fisheries data. (PDF)

S1 Dataset Summarized data. (XLSX)
