# Supplementary material for: Quality of a fished resource: Assessing spatial and temporal dynamics
Source: PLoS One. 2018 Jun 6;13(6):e0196864. doi: 10.1371/journal.pone.0196864 (PMC5991392; doi:10.1371/journal.pone.0196864)
Supplement: S1 Appendix — (PDF) [file pone.0196864.s001.pdf]

S1 Appendix. Sea urchin processor quality evaluation and seasonal data.

The California Sea Urchin Commission (www.calurchin.org) developed grade names and guidelines to assist in standardizing uni quality within the industry. "California Gold" is the highest quality, or *grade A* uni, and the gonads have a bright color, firm texture, and are fresh and intact. Sea urchins are graded as "Premium California," or *grade B* uni, if the gonads are slightly duller in color, firm texture, fresh and mostly intact. Grade A and grade B uni are sold fresh to distributors and restaurants, largely to be consumed raw. "Select California" is also known as *vana* (also spelled as *wana*, the Hawaiian word for urchin), or grade C; gonads from these sea urchins vary in color (often darker), have softer texture, are watery and may be broken in pieces. Vana is usually shipped frozen and makes up about 5-10% of the gonads extracted (D. Rudie, pers. obs.). For each trip per boat, processors in California categorize the sea urchin product within these three grades. Recently retail values (USD) of grade A, B, and vana are approximately $110-225/kg, $22-168/kg, and $7-55/kg (or, $50-102/lb, $10-76/lb, and $3-25/lb), respectively as of 2015, and around $57/kg, $13/kg, and $2/kg (or, $26/lb, $6/lb, and $1/lb) go to the fishermen per grade, respectively.

In recent years, grade A is mostly sold in the USA, and grade B is largely exported to Japan (D. Rudie, pers. obs.). Currently about 80-95% of grade A remains in the domestic market and the rest largely is shipped to Japan for auction. However, 30 years ago about 95% of grades A and B was exported to Japan. In the past decade California supplied about 80% of the domestic market for uni, but more than 50% of the annual harvest (by volume) was exported (D. Rudie, pers. obs.). However, recently (2016-2017) less than 50% of the domestic market is supplied by the California fishery and less than 10% of the annual harvest (by volume) is exported (D. Rudie, pers. obs). By volume California exports around 50% of the landings (mostly to buyers in Japan) making up about 10-15% of Japanese supply. However, by value only about 25-35% is exported (D. Rudie, pers. obs.).

The sea urchin industry also uses these grades to calculate the overall gonad *yield* to convey a general measure of quality per fishing trip. Yield is often calculated as the percentage of high-grade uni (grades A and B) that was extracted from a fisherman's load. In other words, from the entire load weighed at the dock, yield is the percentage of uni which is most valuable to the industry. While yield (grades A+B, based on instantaneous qualification) is an important factor the industry uses to set the price of a batch of sea urchins, we investigated whether GSI (based on a quantitative index) can adequately capture the variability in price.

We expected yield to be less than GSI because gonad weight measured by processors is often lower and total weight is often higher for several reasons (some referenced in [1]):

(1) processor gonad weight does not include the vana or any pieces of gonad that were discarded due to low quality or breakage;

(2) processors use the total weight of the sea urchins as the entire load weighed at the dock which occasionally could include undersized sea urchins, purple sea

urchins, debris from the sea urchin nets (e.g. other organisms, small rocks attached to the sea urchins, or pieces of kelp);

(3) to calculate GSI, we weighed each whole sea urchin (on an individual basis) typically 12-24 hours after collection, when some water weight may have been lost from the sea urchin; and

(4) processors soak gonads in an anhydrous potassium alum, $KAl(SO_4)_2$, solution that will draw some of the water weight out of the gonads. In addition, after soaking in the solution, gonads are placed on towels to soak up any dripping water. Whereas, when we calculated GSI, we did not use towels to soak up any dripping water, as that methodology would have resulted in the towel also soaking up any spawn if present.

The seasonal processor data showed a fall peak in price per kg (USD) and in percent grade A and B of total landings, which roughly matches the November peak GSI (Fig A). In addition, we examined processor price records, since in recent years a practice of reporting an arbitrary price in the California Department of Fish and Wildlife (CDFW) landing receipts has resulted in inaccurate CDFW data. Since our analyses with both processor and CDFW price datasets were similar, we used CDFW price data since the sample size was larger. Processor price was the lowest in February. Finally, we examined the pattern of quality across season by calculating the percent grade A and B gonad extracted from the total landings. Percent grade B of the total landings was lowest in April, coinciding with the GSI minimum both in port and processor samples.

References

1. Kato S, Schroeter SC. Biology of the red sea urchin, *Strongylocentrotus franciscanus*, and its fishery in California. Mar Fish Rev. 1985;47: 1–20. Available: http://spo.nmfs.noaa.gov/mfr473/mfr4731.pdf

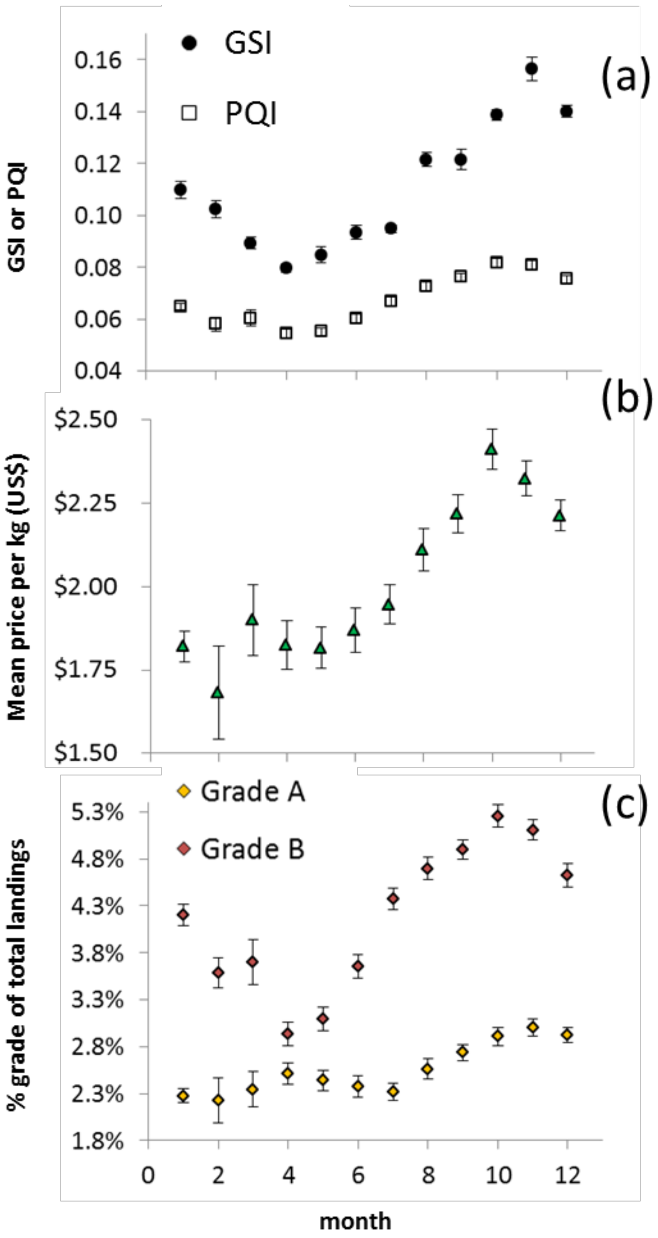

S1 Fig A. Monthly red sea urchin gonadosomatic index and processor data. Monthly (a) mean port-sampling gonadosomatic index (GSI) and processor quality index (PQI), and monthly data from red sea urchin data from a processor (b) mean price per kg (USD) and (c) percent grade A and B gonad weight of total landings weight. Error bars for price, GSI, PQI, and percent grade show one standard error (note: many error bars are smaller than marker size). Processor quality index does not include the weight of the *vana* or any gonads that were discarded due to low, unmarketable quality (because these data were unavailable).
