# Supplementary material for: Quality of a fished resource: Assessing spatial and temporal dynamics
Source: PLoS One. 2018 Jun 6;13(6):e0196864. doi: 10.1371/journal.pone.0196864 (PMC5991392; doi:10.1371/journal.pone.0196864)
Supplement: S2 Appendix — (PDF) [file pone.0196864.s002.pdf]

S2 Appendix. Red sea urchin gonadosomatic index: temporal and spatial details.

S2 Table A. Summary of port sampling of red sea urchins. Mean gonadosomatic index (GSI) per month, sample size of boats (N_b) and individual red sea urchins (N_u), and one standard error (SE), which was calculated using N_u. Total shows average GSI across all months and the sum of each sample size type from San Miguel (SMI), Santa Rosa (SRI), and Santa Cruz Islands (SCI).

	SMI				SRI				SCI†				Overall	
**Month**	**GSI**	**N_b**	**N_u**	**SE**	**GSI**	**N_b**	**N_u**	**SE**	**GSI**	**N_b**	**N_u**	**SE**	**GSI**	**SE**
**1**	0.107	3	40	0.005	0.112	5	55	0.005	.		0	.	0.110	0.003
**2**	0.096	1	10	0.009	0.100	3	30	0.006	0.105	3	61	0.005	0.102	0.003
**3**	0.088	6	61	0.003	0.088	11	126	0.003	0.098	2	23	0.005	0.089	0.002
**4**	0.066	7	80	0.003	0.087	12	131	0.003	0.083	3	30	0.003	0.080	0.002
**5**	0.066	2	28	0.005	0.095	6	76	0.004	0.076	2	25	0.007	0.085	0.003
**6**	0.092	2	20	0.006	0.089	13	130	0.003	0.107	5	50	0.006	0.093	0.003
**7**	0.097	11	110	0.003	0.094	20	200	0.002	0.094	13	130	0.003	0.095	0.002
**8**	0.128	7	70	0.005	0.119	10	104	0.004	0.120	9	88	0.005	0.121	0.003
**9**	0.135	11	116	0.004	0.091	3	30	0.010	0.089	2	20	0.008	0.121	0.004
**10**	0.144	17	170	0.003	0.142	16	162	0.003	0.119	7	70	0.005	0.139	0.002
**11**	0.157	8	80	0.005	0.156	4	39	0.008	.		0	.	0.157	0.005
**12**	0.150	13	170	0.003	0.130	17	182	0.003	0.142	4	39	0.008	0.140	0.002
**Total**	0.111	88	955		0.109	120	1265		0.103	50	536		0.113	0.001

† There were no sea urchins sampled from fishermen at SCI during January and November.

S2 Table B. Summary of red sea urchin port sampling within-island monthly gonadosomatic indices. Summary of red sea urchin port sampling (2009-2011) from San Miguel (SMI), Santa Rosa (SRI), and Santa Cruz Islands (SCI): within-island and overall month-to-month mean gonadosomatic index (GSI) differences: (a) ANOVA results and (b) post-hoc using Student's t (levels not connected by the same letter are significantly different). The six months with the lowest levels are highlighted in gray.

(a)

	**n**	**R^2**	**F Ratio**	***P***
**SMI**	955	0.369	50.1	< 0.0001
**SRI**	1265	0.258	39.7	< 0.0001
**SCI**	536	0.161	11.2	< 0.0001
**Overall**	2756	0.272	93.3	< 0.0001

(b)

SMI								
**Mo**	**GSI**							
**11**	A							0.1568
**12**	A	B						0.1499
**10**		B	C					0.1439
**9**			C	D				0.1349
**8**				D				0.1280
**1**					E			0.1068
**7**					E	F		0.0966
**2**					E	F		0.0963
**6**					E	F		0.0917
**3**						F		0.0879
**5**							G	0.0664
**4**							G	0.0663

SRI							
**Mo**	**GSI**						
**11**	A						0.1561
**10**		B					0.1416
**12**			C				0.1302
**8**				D			0.1187
**1**				D	E		0.1120
**2**					E	F	0.0998
**5**						F	0.0947
**7**						F	0.0941
**9**						F	0.0909
**6**						F	0.0886
**3**						F	0.0884
**4**						F	0.0869

SCI							
**Mo**	**GSI**						
**12**	A						0.1419
**8**		B					0.1195
**10**		B					0.1189
**6**		B	C				0.1067
**2**			C	D			0.1046
**3**			C	D	E		0.0982
**7**				D	E		0.0939
**9**			C	D	E	F	0.0893
**4**					E	F	0.0834
**5**						F	0.0757

Overall									
**Mo**	**GSI**								
**11**	A								0.1566
**12**		B							0.1399
**10**		B							0.1386
**9**			C						0.1215
**8**			C						0.1215
**1**				D					0.1098
**2**				D	E				0.1023
**7**					E	F			0.0947
**6**					E	F			0.0934
**3**						F	G		0.0894
**5**							G	H	0.0849
**4**								H	0.0796

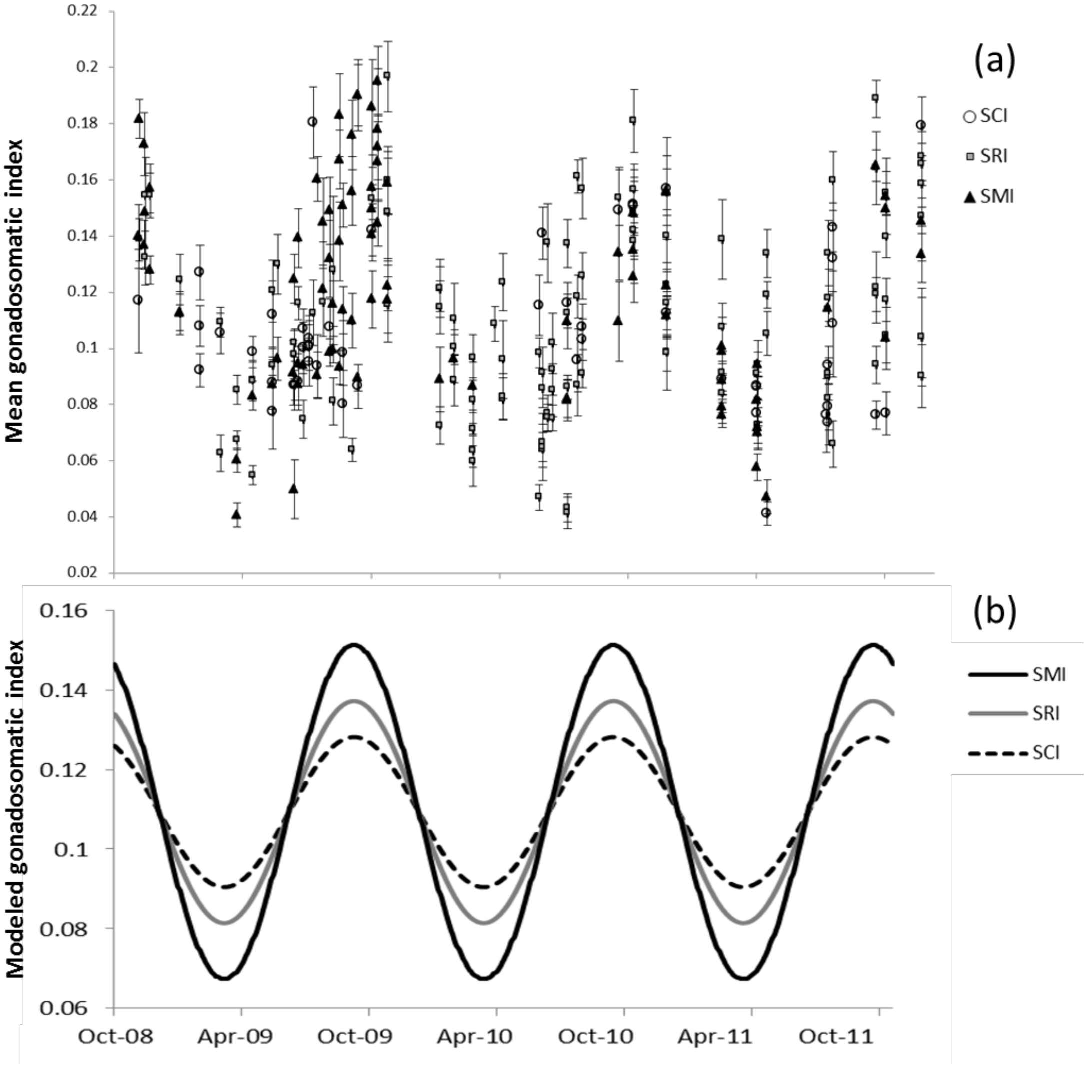

S2 Fig A. Red sea urchin gonadosomatic index per vessel and modeled data. (a) Mean gonadosomatic index (GSI) per vessel sampled from red sea urchins landed at the port of Santa Barbara from December 2008 to December 2011 per island from west to east: San Miguel Island (SMI), Santa Rosa Island (SRI), and Santa Cruz Island (SCI); error bars show one standard error. (b) Modeled GSI using a sinusoidal function for viewing purposes only.
