# Supplementary material for: Quality of a fished resource: Assessing spatial and temporal dynamics
Source: PLoS One. 2018 Jun 6;13(6):e0196864. doi: 10.1371/journal.pone.0196864 (PMC5991392; doi:10.1371/journal.pone.0196864)
Supplement: S3 Appendix — (PDF) [file pone.0196864.s003.pdf]

S3 Appendix. Monthly variation of red sea urchins: spawning and fisheries data.

S3 Table A. Monthly percent spawning port-sampled red sea urchins per island and overall. (a) The presence of spawning in sea urchins during laboratory dissections from port-sampled red sea urchins per month (2009-2011) from San Miguel (SMI), Santa Rosa (SRI), and Santa Cruz Islands (SCI) was recorded. Spawning levels are highlighted according to the degree of spawning: 50% or > is light gray, 60% or > is medium gray, and 70% or > is black. Note: presence of spawning was indicated when active spawning was observed during dissections. Some individuals at the spawning stage may have gone undetected, thus these percentages may be biased downward. (b) ANOVA results comparing differences in percent spawning levels per month within each island and overall within the entire region. (c) Post hoc Student's t-test showing monthly differences in spawning levels within the region (levels not connected by the same letter are significantly different).

(a)

Month	Percent spawning per island			Overall percent spawning
	SMI	SRI	SCI	
1	58	79	--	69
2	10	20	56	29
3	65	48	85	63
4	61	56	53	57
5	36	57	17	37
6	60	33	56	47
7	23	31	45	33
8	24	22	31	26
9	23	17	0	13
10	37	31	23	30
11	36	29	--	33
12	71	59	70	66

(b)

Region	SMI	SRI	SCI	Overall
R^2	0.78	0.66	0.69	0.51
F-ratio$_{DF}$	$F_{11,21}$	$F_{11,26}$	$F_{9,19}$	$F_{11,68}$
	3.25	2.70	2.46	5.39
P	0.0369	0.0383	0.0881	< 0.0001

(c)

Overall					
**Month**					**Mean percent spawning**
**1**	A				69
**12**	A				66
**3**	A				63
**4**	A	B			57
**6**	A	B	C		47
**5**		B	C	D	37
**7**			C	D	33
**11**		B	C	D	33
**10**			C	D	30
**2**			C	D	29
**8**				D	26
**9**				D	13

SMI						
**Month**						**Mean percent spawning**
**12**	A					71
**3**	A	B				65
**4**	A	B	C			61
**6**	A	B	C	D		60
**1**	A	B	C			58
**10**		B	C	D	E	37
**11**	A	B	C	D	E	36
**5**		B	C	D	E	36
**8**			C	D	E	24
**9**			C	D	E	23
**7**				D	E	23
**2**					E	10

SRI					
**Month**					**Mean percent spawning**
**1**	A				79
**12**	A	B			59
**5**	A	B	C		57
**4**	A	B	C		56
**3**	A	B	C	D	48
**6**		B	C	D	33
**7**		B	C	D	31
**10**		B	C	D	31
**11**		B	C	D	29
**8**				D	22
**2**		B	C	D	20
**9**			C	D	17

SCI				
**Month**				**Mean percent spawning**
**3**	A			85
**12**	A	B		70
**6**	A	B	C	56
**2**	A	B	C	56
**4**	A	B	C	53
**7**	A	B	C	45
**8**		B	C	31
**10**			C	23
**5**			C	17
**9**			C	0

S3 Table B. Monthly variation in red sea urchin mean gonad yield from Fort Bragg (1991-1992). Monthly variation in red sea urchin mean gonad yield (similar to gonadosomatic index); post-hoc using Student's t (levels not connected by the same letter are significantly different) from ANOVA test ($F_{10,496} = 6.84$, $P < 0.0001$, $R^2 = 0.123$). Data acquired from the California Department of Fish and Wildlife originally from processors in 1991-1992 from Fort Bragg, California (Kalvass, pers. comm.; [1]). This region is where management examined price data (and gonadosomatic index data) to set reduced effort during the months where prices were the lowest due to low quality. The timeframe examined by management was 1991-1992. The six months with the lowest yield are highlighted in gray, however there is a lot of overlap in yield among months. July was not included, as the fishery is closed during this month in this location during these years.

**Month**							**Mean gonad yield**
**1**	A	B					10.62
**2**	A						10.61
**3**	A						10.52
**12**	A	B	C				9.87
**4**	A	B	C				9.70
**10**		B	C				9.54
**11**	A	B	C	D			9.40
**9**			C	D			9.11
**5**			C	D			8.84
**8**				D	E		8.14
**6**					E		7.54

S3 Table C. Monthly variation in red sea urchin mean price per kilogram from Fort Bragg (1991-1992). Post-hoc using Student's t (levels not connected by the same letter are significantly different) from ANOVA test ($F_{11,8976} = 109.4$, $P < 0.0001$, $R^2 = 0.118$). California Department of Fish and Wildlife data summarized from 1991-1992 from the port of Fort Bragg, in northern California. This region is where management examined price data (and gonadosomatic index data) to set reduced effort during the months where prices were the lowest due to low quality. The timeframe examined by management was 1991-1992. The six months with the lowest prices are June through October and January (highlighted in gray).

**Month**									**Mean price per kg (USD)**
**12**	A								2.33
**3**		B							1.83
**2**		B							1.82
**4**			C						1.59
**11**			C	D					1.49
**5**			C	D					1.46
**1**				D	E				1.36
**10**					E	F			1.24
**9**						F	G		1.19
**6**							G	H	1.07
**8**								H	0.95
**7**					E	F	G	H	0.51

S3 Table D. Monthly variation in red sea urchin mean price per kilogram from Fort Bragg (2002-2011). Post-hoc using Student's t (levels not connected by the same letter are significantly different) from ANOVA test ($F_{11,12527} = 70.5$, $P < 0.0001$, $R^2 = 0.058$). California Department of Fish and Wildlife data summarized from 2002-2011 from commercial red sea urchins landing into the port of Fort Bragg. The seven months with the lowest prices are March through September (highlighted in gray).

**Month**						**Mean price per kg (USD)**
**12**	A					1.67
**1**		B				1.54
**11**			C			1.40
**2**			C			1.40
**10**			C			1.37
**3**				D		1.32
**9**				D		1.32
**4**				D		1.29
**7**					E	1.23
**6**					E	1.21
**5**					E	1.19
**8**					E	1.18

S3 Table E. Monthly variation in red sea urchin mean price per kilogram from the Channel Islands. Post-hoc using Student's t (levels not connected by the same letter are significantly different) from ANOVA test ($F_{11,117359} = 52.3$, $P < 0.0001$, $R^2 = 0.0048$). California Department of Fish and Wildlife data summarized from 2002-2011 from the 14 blocks surrounding the Channel Islands. The six months with the lowest prices are February through May and August and November (highlighted in gray).

Month									Mean price per kg (USD)
12	A								$1.63
7		B							$1.59
10		B	C						$1.58
9		B	C	D					$1.57
1			C	D	E				$1.56
6				D	E				$1.55
11					E	F			$1.54
8						F			$1.52
4							G		$1.46
2							G		$1.46
3							G	H	$1.44
5								H	$1.42

References

1. Kalvass PE, Hendrix JM. The California red sea urchin, *Strongylocentrotus franciscanus*, fishery: Catch, effort, and management trends. Mar Fish Rev. 1997;59: 1–17. Available: http://spo.nmfs.noaa.gov/mfr592/mfr5921.pdf
